# Novel Hypoxanthine Guanine Phosphoribosyltransferase Gene Mutations in Saudi Arabian Hyperuricemia Patients

**DOI:** 10.1155/2014/290325

**Published:** 2014-07-09

**Authors:** Mohammed Alanazi, Abdulrahman Saud Al-Arfaj, Zainularifeen Abduljaleel, Hussein Fahad Al-Arfaj, Narasimha Reddy Parine, Jilani Purusottapatnam Shaik, Zahid Khan, Akbar Ali Khan Pathan

**Affiliations:** ^1^Department of Biochemistry, College of Science, King Saud University, Riyadh 11451, Saudi Arabia; ^2^Department of Medicine, King Saud University Hospital, Riyadh 11426, Saudi Arabia; ^3^Department of Medical Genetics, Faculty of Medicine, Umm Al-Qura University, Makkah 21955, Saudi Arabia

## Abstract

Over the past decade, a steady increase in the incidence of HPRT-related hyperuricemia (HRH) has been observed in Saudi Arabia. We examined all the nine exons of HPRT gene for mutations in ten biochemically confirmed hyperuricemia patients, including one female and three normal controls. In all, we identified 13 novel mutations in Saudi Arabian HPRT-related hyperuricemia patients manifesting different levels of uric acid. The Lys103Met alteration was highly recurrent and was observed in 50% of the cases, while Ala160Thr and Lys158Asn substitutions were found in two patients. Moreover, in 70% of the patients ≥2 mutations were detected concurrently in the HPRT gene. Interestingly, one of the patients that harbored Lys103Met substitution along with two frameshift mutations at codons 85 and 160 resulting in shortened protein demonstrated unusually high serum uric acid level of 738 *μ*mol/L. Two of the seven point mutations that resulted in amino acid change (Lys103Met and Val160Gly) were predicted to be damaging by SIFT and Polyphen and were further analyzed for their protein stability and function by molecular dynamics simulation. The identified novel mutations in the HPRT gene may prove useful in the prenatal diagnosis and genetic counseling.

## 1. Introduction

Multiple factors including genetic, food habits, age, sex, medication, and environment play a vital role in high serum urate levels [1–5]. Evidence suggests that people who changed their lifestyle including adoption of westernized diet [6–8] are easily prone to elevated levels of serum uric acid. Hyperuricemia is caused by food which is rich in purines, high alcohol intake, fructose ingestion, and obesity all of which accelerates ATP degradation to AMP which is a precursor of uric acid. Although multiple etiologies have been proposed over the last decade, hereditary renal disorders with rare mutant allele cause distinct clinical hyperuricemia. One of the important genetic factors although rare is the mutations involved in the phosphoribosyltransferase deficiency and PRPP [phosphoribosyl pyrophosphate] synthetase super activity. Hypoxanthine guanine phosphoribosyltransferase (HPRT) deficiency has been implicated in hyperuricemia. HPRT is the most important enzyme involved in the purine salvage pathway, the function of which is to recycle hypoxanthine and guanine into readily available nucleotide pool [9]. HPRT is encoded by a single gene located on the X-chromosome (Xq26-27) with nine exons that transcribe into a 1.6 kb mRNA encoding a protein of 218 amino acids [10]. Severity of HPRT-related diseases depends on the extent of enzyme inactivity. A partial deficiency of HPRT leads to Kelley-Seegmiller syndrome (MIM300323), which results in hyperuricemia, hyperuricaciduria, uric acid nephrolithiasis, and precocious gout arthritis [11]. While many different clinical phenotypes manifesting different levels of hyperuricemia are reported, the most severe form is the classical Lesch-Nyhan disease (LND) and the least is characterized by hyperuricemia without any neurological or behavioral abnormality and termed as HPRT-related hyperuricemia (HRH) [12, 13]. In between these two extremes are the phenotypes expressing hyperuricemia but with varying degrees of neurobehavioral abnormality designated as HPRT-related neurological dysfunction (HRND). The lack of mutational hot-spot for HPRT gene gives rise to the observed genetic heterogeneity in HPRT deficient patients. Many different mutations including deletions, insertions, duplications, abnormal splicing, and point mutations in all the nine exons have been identified at the HPRT gene locus. Lists of more than 600 different HPRT mutations are posted in the research sections at http://www.lesch-nyhan.org/.

The mutational status of HPRT gene in Saudi Arabian hyperuricemia patients is still obscure. Hence, in this study we sequenced all the nine exons of HPRT gene and identified 13 novel HPRT mutations in Saudi Arabian patients manifesting different degrees of hyperuricemia, including a rare affected female patient. Further, computational based methods were utilized to predict the effect of identified mutation on the structure and function of HPRT enzyme. Computational methods are increasingly being used to understand the dynamic behavior of wild type and mutant proteins at atomic level [14, 15]. To explore the possible associations between genetic mutation and varying degrees of hyperuricemia, the mutated amino acids stability was analyzed using SIFT, Polyphen, and PoPMusic, and its function using screening for nonacceptable polymorphisms (SNAP). We further examined the native and mutant protein structures for solvent accessibility and secondary structure analyses. Additionally, molecular dynamics (MD) simulations were performed to investigate the motions of the native and the mutant particles under the influence of both external and internal forces. The overall results provide structure-activity relationship for both the wild type and the mutant proteins.

## 2. Materials and Methods

### 2.1. Study Samples

Patients (*n* = 10) with biochemically confirmed hyperuricemia attending the rheumatology clinics of the King Khalid University Hospital, Riyadh, served as cases and normal healthy males (*n* = 2) and females (*n* = 1) with no history of hyperuricemia were considered as controls. Cases with uric acid overproduction were designated HPRT-related hyperuricemia. All the selected patients showed hyperuricemia with varying degrees (serum uric acid, Table 1) but none of them were diagnosed with neurological abnormalities or self-injurious behavior. The study was approved by the institutional review board of the King Khalid University Hospital and informed consent was obtained from each study participant.

### 2.2. DNA Extraction and Sequencing

Approximately 3 mL of blood was collected in sterile tubes containing ethylenediaminetetraacetic acid (EDTA) from all subjects enrolled in the study. Genomic DNA was isolated from blood samples using QIAmp kit (QIAmp DNA blood Mini Kit, Qiagen, Valencia, CA) following the manufacturer's instructions. After extraction and purification, the DNA was quantitated on a NanoDrop 8000, to determine the concentration, and its purity was examined using standard A260/A280 and A260/A230 ratios (NanoDrop 8000) [16]. Primers were designed to amplify all nine HPRT gene exons and flanking intronic sequences by polymerase chain reaction (PCR) (see Supplementary Table 1 in Supplementary Material available online at http://dx.doi.org/10.1155/2014/290325), on the basis of the known genomic sequence (accession number NM 000194). PCR products were purified by ethanol precipitation after ExoSAP (GE Health Bio-Science Ltd.) treatment and sequenced on the sense and antisense strands utilizing the DYEnamic ET Terminator Cycle Sequencing Kit (Amersham Biosciences). The sequencing reactions were analyzed on an automatic* MegaBACE 1000* DNA sequencer (GE Health Bio-Science Ltd).

### 2.3. Predicting the Functionality of the Nonsynonymous Mutations

In the present study we have identified 13 novel (7 nonsynonymous, 1 synonymous, 4 frameshift, and a 20 bp deletion) mutations (Table 2) in Saudi Arabian HPRT-related hyperuricemia patients, including a female subject. As the observed mutations were novel, we used SIFT and Polyphen programs to predict the possible impact of an amino acid substitution on the structure and function of HPRT protein. SIFT (http://sift.jcvi.org/) is a sequence homology based tool that predicts variants as neutral or deleterious using normalized probability score. Variants at position with normalized probability score less than 0.05 are predicted to be deleterious and score greater than 0.05 are predicted to be neutral [17]. PolyPhen 2.0 (http://genetics.bwh.harvard.edu/pph2/index.shtml) utilizes a combination of sequence and structure based attributes and uses naive Bayesian classifier for the identification of an amino acid substitution and the effect of mutation. Scores are predicted to affect protein function and are tabulated in Table 2. The results obtained from SIFT and Polyphen were further validated using SNPnexus program (http://snp-nexus.org/). SNPnexus allows single queries using dbSNP identifiers or chromosomal regions for annotating known variants. SNPnexus summarizes (Table 2) any related information from genetic association studies of complex diseases and disorders available from the genetic association database (GAD) [18] and overlaps with genomic structural variability [19–27], dbSNP [28], and HapMap [29] genotype and allele frequency.

### 2.4. Protein Modeling for the Altered Amino Acid

Two of the 13 mutations which were found to be damaging from SIFT and Polyphen results and which were common among patients were further examined in silico for 3D structural effects. We used X-ray diffraction structure of human hypoxanthine guanine phosphoribosyltransferase (1BZY) available at PDB database as the reference model. The native protein (1BZY) structure was then compared with the predicted mutant structures of all the amino acid variations and their solvent accessibility including secondary structures was modeled using molecular dynamics (MD) simulation.

Prediction of the three-dimensional structure of the two variants (Lys => Met at position 103 and Val => Gly at position 160) of the HPRT gene located on chromosome X was performed using I-TASSER server [30]. The server predicts the lowest energy conformation for all the amino acid variations and returns five best models according to* C*-score.* C*-score is a confidence rating factor in I-TASSER to estimate the overall quality of the predicted model. TM (topological similarity of the two structures) scores below 0.3 after TM alignment are selected for homology modeling (threading template alignments).

### 2.5. Predicting the Stability of the Altered Amino Acids

Stability of the mutant protein is checked using Discovery Studio 2.5 (DS Modeling 2.5, Accelrys Inc., San Diego, CA, USA) a commercially available software used at* DNA LABS INDIA*, Hyderabad, India. Residue scanning for predicting protein stability was performed using the mutant secondary structure. Residue scanning was used to identify polar and neutral residues to analyze protein stability and solvent accessibility which can later be fixed for errors. Additionally, we also used screening for nonacceptable polymorphisms (SNAP) [31] and PoPMuSiC [32] to predict the stability of the mutations for consistency. SNAP is used for the prediction of impact of missense mutation based on neural network and improved machine-learning methodologies. For each mutant, SNAP returns three values: the binary prediction (neutral/nonneutral), the reliability index (RI, range 0–9), and the expected accuracy that estimates accuracy on a large dataset at the given RI. SNAP scores differ from −100 (strongly predicted as neutral) to 100 (strongly predicted to alter function); the distance is directly related to the binary determination boundary (0), which measures the reliability of the impact. A damaging signal corresponds to a mutation that is predicted as being stabilizing. SNAP scores pertain to extra functional effects whereas PoPMuSiC evaluates the changes in stability of a given protein or peptide under single-site mutations (Table 3), on the basis of the protein's structure. Additionally, independent evaluations for predicting stability variations upon mutation were also performed using I-Mutant and MutPred (data not shown).

### 2.6. Model Verification

The quality check of the 3D models (mutant) was assessed by ProSA-web. ProSA-web calculates the energy profiles (*z*-score) for modeled structure by using molecular mechanics force field. The results are predicted as *z*-score which measure the total energy deviation of the wild type and the mutant protein. Based on the deviation of the *z*-score the models can be accepted or rejected. *z*-Score plot can also be used for better interpretation of the *z*-score of a specified protein.

### 2.7. Molecular Dynamics Simulation

The homology modeled three-dimensional structure of HPRT protein submitted to protein databank [PDB: 1BZY] was used to study the effect of mutated residue. Structures deduced for HPRT harboring mutations Lys103Met and Val160Gly identified in our samples were utilized for the analyses. Biopolymer module implemented in InsightII (Accelrys Inc., San Diego, CA, USA) was used to modify the mutated residues from the InsightII fragment library. Using the same module, hydrogen atoms were added to both wild type and mutated protein structures at pH 7.0.* CHARMM force fields* [33] were applied to both structures. Further, a series of energy minimization steps were performed on both protein structures by InsightII/Discover (Accelrys Inc., San Diego, CA, USA) using the following protocol. (a) In the first step of minimization, all the heavy (all nonhydrogen) atoms were constrained; the hydrogen atoms were allowed to minimize by steepest decent algorithm until the maximum derivative (|dE/dr|) of the system was <1 kcal/(mole$\cdot \overset{´}{Å}$). (b) This step was followed by another steepest descent minimization with the same parameter as in step (a), but constraining the protein backbone atoms and relaxing all other atoms of the molecule. (c) In the final step, the protein molecule was minimized by conjugate gradient method with the backbone atom fixed and allowing all other atoms to relax until the maximum derivative was <0.01 kcal/(mole$\cdot \overset{´}{Å}$). The deviation between the two structures is evaluated by their RMS values which could affect stability and functional activity (Supplementary Tables 2–4, Supplementary Figures 1–3). Structure analysis of protein after energy minimization of protein structure was analyzed using Discovery Studio 2.5 (DS Modeling 2.5, Accelrys Inc., San Diego, CA, USA).

## 3. Results

### 3.1. Novel Mutations of HPRT

A total of 10 patients diagnosed with hyperuricemia based on clinical and laboratory findings were included in the present study (Table 1). All the patients were examined by a rheumatologist and informed consent was obtained from patients and relatives for genetic and biochemical studies. Patients' ages ranged from 26 to 83 years (mean age 54 years). Three healthy controls without metabolic dysfunction, aged 26 to 86 years, were also examined for serum uric acid levels and included in the study. One healthy control was a sibling of an affected patient included in the study group. Although the patients presented were hyperuricemic, none of them showed any self-injury or neurological problems. No mutations were detected in the HPRT gene in the controls except a synonymous substitution at codon 160 in the related sibling.

Results of mutation analysis of all the 9 exons of HPRT gene are shown in Table 2. We identified 13 novel (7 nonsynonymous, 1 synonymous, 4 frameshift, and a 20 bp deletion) mutations in Saudi Arabian HPRT-related hyperuricemia patients. Out of the 13 mutations, seven were missense leading to single amino acid substitutions, one was synonymous, three were nonsense point mutations that lead to premature stop codons resulting in truncated protein, and a three-base pair deletion leading to peptide shift and a large deletion of 20 bp at chromosomal position X: 133624250-133624269 between exon 5 and exon 6 were observed. Surprisingly, none of the mutations were reported earlier in any other populations. Lys103Met substitution in exon 3 was highly prevalent and was detected in 50% of the patients. A summary of the mutational analysis and their occurrences in patients are presented in Tables 1 and 2. Although there was no correlation between the type or number of mutations and serum uric acid level (Table 1), one patient, exhibiting a missense mutation (Lys103Met) along with an insertion (329InsG) and a 20 bp deletion in the intronic region between exon 5 and exon 6, suffered from very high uric acid levels (>700 *μ*mol/L). Further, no mutation in the entire nine-exonic region of HPRT gene was detected in patient number 3 which has significantly high serum uric acid level of 564 *μ*mol/L (Table 1). As the observed mutations were novel, we tried to predict the structural and functional consequences of the amino acid substitutions using SIFT and Polyphen. These predictions were further tested for disease association using SNPnexus. Out of the 13 mutations that were observed, 3 (Leu85Val, Lys103Met, and Val160Gly) were found to be damaging from both SIFT and Polyphen predictions (Table 2). Point mutation at codon 85 resulted in frameshift; hence we considered HPRT with amino acid Lys103Met and Val160Gly substitutions for SIFT, Polyphen, and MD simulation analyses. For three of the mutations (Val160Val, Val160Arg, Val, Ala160Ala, Ser) SIFT and Polyphen could not generate a prediction. Surprisingly, all the 12 mutations showed an association with Lesch-Nyhan syndrome according to SNPnexus (Table 2).

### 3.2. Amino Acid Alterations on Protein Stability

The results showed an excessive-folding free energy (ΔΔ*G* = −0.04 for Lys103Met and 3.23 kcal/mol for Val160Gly) for the two mutations that were predicted to be damaging. Val160Gly was predicted to be destabilizing, whereas Lys103Met was predicted to be stabilizing. SNAP predicted Val160Gly and Lys103Met to be nonneutral. Substitution of Lys103Met and Val160Gly resulted in a slight worsening of ProSA-web *z*-score, from −8.3 to −8.04 (Figure 2(d)), the total energy deviation is −2.74 which might have a very unfavorable change on the protein structure and function. Results from independent evaluation programs like I-Mutant and Mutpred (data not shown) also hinted that the protein stability decreased upon mutations at positions 103 and 160 of HPRT.

### 3.3. MD Simulations of the Native and Mutant Residues (Lys103Met and Val160Gly)

**Figure 1:**
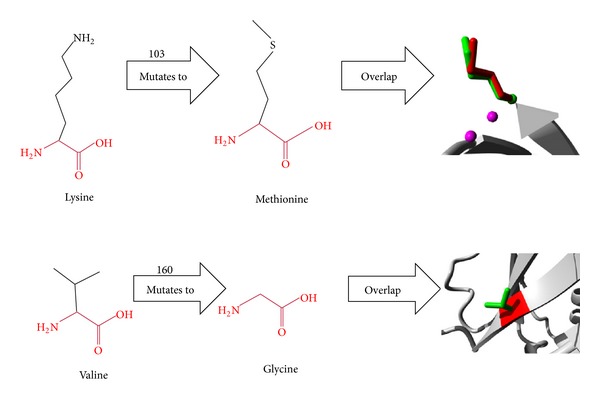
Schematic structure of the wild type and mutant (Lys103Met and Val160Gly) amino acids.

The 3D structure of HPRT has already been deduced (PDB ID: 1BZY). Annotations about this protein were obtained from Uniprot-entry P00492. We were interested to examine the functional effects of Lys103Met and Val160Gly substitutions in HPRT.  Figure 1 shows the schematic structures of the wild type (left) and the mutant (middle) amino acids. The backbone, which is the same for each amino acid, is colored in red. The side chain, unique for each amino acid, is colored in black. Each amino acid has its own specific size, charge, and hydrophobicity-value. The mutant residues (Glycine and Methionine) are smaller than the wild type (Valine and Lysine) residues. The wild type residue (Lysine) at codon 103 is positively charged while the mutant residue (Methionine) is neutral and more hydrophobic than the wild type residue. Both wild type residues are not conserved at their respective positions when compared with other homologous sequences. However, the other amino acids found in homologous sequences at these positions are not similar to the mutants that we observed. Thus, these mutations may alter the structure and have deleterious effect on the function of HPRT.

The probability that a mutation will cause disease tends to be higher when the missense mutation was within a functionally important region. MD simulation was essential to make predictions of the effects of novel HPRT mutations, opening possibilities for exploring properties of the molecular system under investigations that are less accessible by conventional experimental methods. For MD simulations, the HPRT gene structural information was obtained from the PDB database (RCSB PDB) and Human Genome Variation database (HGVBASE). Based on the amino acid sequence and the ORF, the HPRT amino acid structure was submitted to I-TASSER program and the best among the five models based on the* C*-score (−3.452), TM-score (0.34 ± 0.11), RMSD (11.0 ± 4.6 Å), number of decoys (1926), and cluster density (0.0433), 1BZY, was selected as the best match.

**Figure 2:**
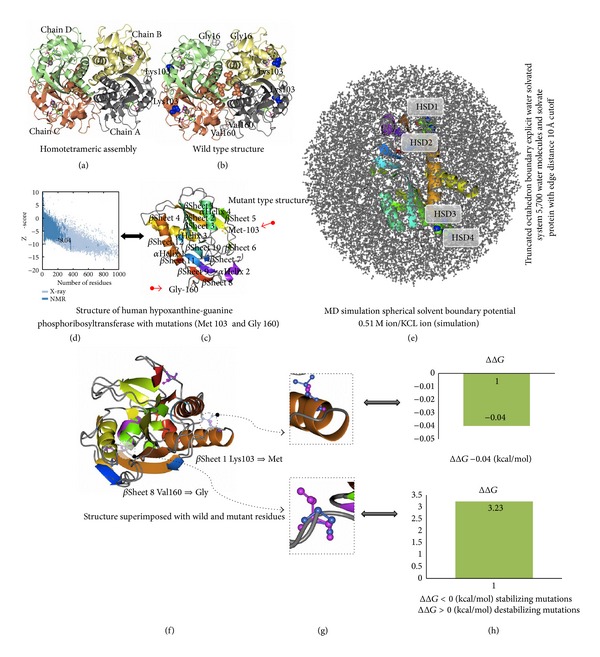
(a) Ribbon diagram of HPRT protein showing all the four side chains A, B, C, and D. (b) Location of mutations (K103 M and V160G) identified in the HPRT protein of hyperuricemia patients. (c) Structure of human hypoxanthine guanine phosphoribosyltransferase A-chain displaying K103 M and V160G mutations. (d) Stability change of the mutant calculated by ProSA server. (e) Molecular dynamics (MD) simulation showing truncated octahedron boundary explicit water solvated and hydrogen atoms. The visual inspection also allows identifying the side chain of histidine residues (HSD1–HSD4) involved in hydrogen bonding with the surrounding molecules. (f) HPRT structure superimposed with wild and mutant residues with mutations K103 M and V160G enlarged (g). (h) Change in the protein stability for the observed mutations.

The human HPRT gene structure (PDB ID: 1BYZ) having 218 amino acid residues with 4 side chains (A, B, C, and D) was used as a wild type protein (Figures 2(a) and 2(b)). The mutations in the predicted HPRT structure were introduced using Discovery Studio 2.5 (DS Modeling 2.5, Accelrys Inc., San Diego, CA, USA) to observe the altered protein structure and to compare it with the native structure (Figure 2(c)). Initially, all the ligand atoms other than water molecules were deleted from the crystal structure. All molecular modeling simulations were performed by Discovery Studio 2.5 (Accelrys, USA) with CHARMm force field and CFF partial charges used in simulations. The target residues were mutated (Lys103Met and Val160Gly) and the lowest energy rotamer conformations were chosen. The atomic positions were minimized by 200 cycles steepest descent (SD) with the following two conditions, (1) the backbone of the protein which was kept fixed and (2) presence of a 35 Å water sphere generated around the center of mass of the protein. All water molecules were subsequently removed from the resulting structure and the protein molecule was solvated using “explicit periodic boundary” option where the system was neutralized and solvated in a spherical box of water molecule with a minimum solute-wall distance of 10.0 Å. The entire system was then optimized initially by 200 cycles SD and then by conjugate gradient (CG) till the derivative reached 0.01 kcal/mol/Å (Supplementary Tables 2–4, Supplementary Figures 1–3) using the Particle Mesh Ewald approach for the evaluation of the electrostatic energy term with 10 Å distance cutoff. A similar protocol was also applied for wild type HPRT (PDB ID: 1BZY) structure to relax the crystal packing force for comparison with the mutant in a similar platform (Supplementary Tables 2–4, Supplementary Figures 1–3). The solvate showed successful aqueous solvent accumulation around the predicted structure. The solvate with octahedral shapes of water box fully fitted to solvate the molecule with edge distance 10.0 (Figure 2(e)). The native and the mutant structures were superimposed to observe the structural consequences due to the mutation (Figures 2(f) and 2(g)). The Discovery Studio 2.5 confirmed that the disease associated variants (Lys103Met and Val160Gly) were located in binding site of predicted structure, signifying that the mutation plays an important role and alters its binding efficacy as well as its structural and functional properties (Figure 2(h)).

## 4. Discussion

Tomita-Mitchell and his colleagues [34] reported 600 different point mutations in the HPRT gene. According to the Human Gene Mutation Database, the mutational spectrum of the HPRT locus contains more than 300 mutations and over 165 (76%) were reported to be in the coding region. At least 72% (155 out of 218) of the amino acids in the HPRT monomer are mutable; that is, a substitution is known to give rise to a clinical or thioguanine-selectable phenotype [35]. This large target for mutation may partly be explained by a high degree of evolutionary conservation and partly by the formation of a complex holoenzyme with large dimer-tetramer interfaces that are sensitive to substitutions. However, the reported missense mutations are not evenly distributed among the 218 codons in the HPRT gene. While there are several frequently mutated codons in HPRT gene, as many as 46 codons exist without any known mutations [36]. The nonrandom distribution of mutations and the existence of significant hot spots and “cold spots” suggest that features related to DNA sequence context as well as DNA replication and repair mechanisms contribute to the observed spectrum of mutations in the HPRT DNA. We observed Lys103Met substitution in 50% of the cases, suggesting this location to be a mutational hotspot in our patient population. Isolated hotspots with the same mutation occurring multiple times in unrelated patients have been reported in earlier studies [37]. Previous analyses of the HPRT mutational spectrum [38–45] had revealed structural features in DNA that probably contribute to mutation induction at the HPRT locus, for example, missense mutations due to single base substitutions leading to amino acid substitutions; nonsense point mutations leading to premature stop codons and truncated proteins; deletions leading to the loss of enzyme activity. Importantly, missense mutations have been shown to be overrepresented within or close to several quasipalindromic sequences in HPRT gene that are predicted to form hairpin structures. However, for most of the missense hotspots at the HPRT locus, the underlying molecular mechanisms of mutagenesis remain to be elucidated. In the present study, we sequenced all the nine exons of HPRT gene utilizing primers designed in the flanking intronic regions of each exon. We identified 12 novel mutations in the protein coding region in the Saudi Arabian hyperuricemia patients.

Although we identified several mutations in the intronic as well as untranslated regions, we reported only those mutations that were found in the protein coding sequences. Although the exact mutations identified in our study have not been found in other populations, several studies reported similar mutational profile in the same regions of the HPRT gene; for example, mutations in the exonic regions 3 and 6 were also reported by several groups and these alterations include 312C>A [38], 314A>G [36], 315T>G [46], 325C>T [47], 329C>T [48], 368C>G [49], 370A>C [50], 389T>A [48], 611A>G [51], 610C>G [52], 610C>T [53], 618T>A [39], and 617G>A [52]. In contrast to some genetic diseases in which one or a small number of mutations account for the diseased condition in majority of patients, HPRT deficiencies are caused by multiple different mutations affecting nearly all parts of HPRT gene. Therefore, the identification of mutation in each family with hyperuricemia must be carried out prior to the prenatal diagnosis. We also observed a large deletion (20 bp) in the intronic region between exons 5 and 6 in one of our hyperuricemia patients. Yamada and colleagues also reported such large deletion and translocation in the HPRT gene [50, 54].

Since deficiency in HPRT is severely X-linked, patients are usually limited to males. However, we identified an unusual case of a female HPRT-related hyperuricemia (HRH) patient harboring a nonsynonymous frameshift mutation at 327InsT and 340T>A. Interestingly, this patient also suffers from Churg Strauss syndrome and taking a close look at the pedigree of this patient revealed that her mother suffers from Asthma, and out of 8 sisters and 3 brothers, 5 sisters suffer from Churg Strauss and one brother succumbed to Asthma. Although there are a few reports stating that Allopurinol (a drug to treat hyperuricemia and GOUT) causes drug rash with eosinophilia and systemic symptoms syndrome, a closer look at the molecular aspects of the mechanism involved will be worth investigating. Several other studies also reported mutations in the HPRT gene in female patients; however, the identified mutations were different than those determined in this study [55–57]. The molecular mechanisms responsible for the HPRT deficiency in the first reported female patient were a complete deletion of maternally inherited HPRTallele and inactivation due to abnormal methylation of the paternal HPRTallele [57]. In another instance of a female patient, a nonsense mutation, R51X, was detected in one of the alleles of HPRT, and a decreased normal mRNA expression from the other allele was observed [58]. So far, five female LND patients with partial HPRT deficiency have been reported. The genotypes of these female patients were heterozygous for mutations similar to the carriers, and a nonrandom X-inactivation of the normal allele occurred [45]. There are also reports which showed skewed X inactivation in monozygotic twins, where one of the twins suffered from the disease [45].

In this study, we identified 13 novel HPRT gene mutations in 10 biochemically confirmed hyperuricemia patients of Saudi Arabian ethnicity. As these mutations were not reported in earlier studies, we further characterized the effect of these mutations on the protein function and stability utilizing computational methods such as SIFT, Polyphen, and MD simulations. In a similar study Nguyen and Nyhan [42] reported novel mutations in the human HPRT gene in three unrelated Lesch-Nyhan patients; however, the absorbed and compensated movements of the structural elements in the solvated protein were not studied in detail. A similar insilico and molecular dynamic simulation strategy was performed by Kamaraj and Purohit [59] to screen disease-associated nsSNP in TYRP1 gene and its structural consequences in OCA3. Using similar tools (PolyPhen 2.0, SIFT, PANTHER, I-mutant 3.0, PhD-SNP, SNP&GO, Pmut, and Mutpred) they found that R326H and R356Q are the most deleterious mutations associated with Oculocutaneous albinism type III (OCA3) disease. In order to have a better understanding of pathological outcomes of a mutant protein, its conformational changes with respect to its native protein have to be studied at molecular level. Molecular dynamic simulation (MDS) had been a preferred platform to study the phenotypic effect induced by point mutations by computational biologists [60, 61]. In our study, we for the first time report the solvated model of the HPRT mutated protein which showed very good correlation between the predicted functional and structural effects on one hand and the observed phenotype on the other hand, leaving few or no alternative options.

For most of the changed residues, we could explain the resulting phenotype by possible structural changes in the dimer interfaces, ligand binding site, or protein hydrophobic core. Interestingly, in the case of the two mutations resulting in amino acid substitutions which were found to be damaging by SIFT and Polyphen, the wild type residues were not conserved at these positions when compared with other homologous sequences. The mechanism pertaining the deleterious functional effect caused by the substituted amino acids needs further elucidation. This is the first study examining the HPRT gene in Saudi Arabian hyperuricemia patients. The novel mutations identified in these patients along with the already reported alterations may prove to be useful in genetic counseling as well as prenatal diagnosis of HPRT-related diseases.

## 5. Conclusions

Overall, the unique nature of most identified mutations, their uniform distribution throughout the gene, and the presence of de novo cases confirmed that hyperuricemia is not linked to any major founder mutation at least in the Saudi Arabian population but is rather the consequence of multiple, separate, independent events randomly affecting the HPRT gene. MD simulation was essential to make predictions of the effects of a novel HPRT mutations, opening possibilities for exploring properties of the molecular system under investigation that are less accessible by conventional experimental methods. MD studies also indicate that the HPRT mutation has negligible effect on the protein when it is free in solution with solvated protein condition but the helical wild type structure gradually loses its secondary structure upon simulation and perhaps most importantly, it was found that, in the presence of this novel mutation, the binding surface is significantly altered preventing HPRT from functioning. The results reported herein strengthen the role of the functional and structural impact in HPRT activity and provide useful information for the design of appropriate mutants for future mechanistic studies. Thus, recent advancements in the use of high performance molecular dynamics with very long-range incessant simulation trajectories and in silico simulation studies have opened new doors to understand mutation-induced changes in proteins at atomic level [62].

## Supplementary Material

The supplementary tables and figures show the PCR conditions and primers used to amplify the 9 exonic regions of the Saudi Arabian hyperuricemia patients (Table 1), with energy minimization and molecular dynamic simulations performed for the substituted amino acids Lys103Met and Val160Gly at various minimization cycles using conjugate gradient method (Tables 2–4, Supplementary Figures 1–3).

## Figures and Tables

**Table 1:** Comparison between the mutational spectrum observed in the HPRT gene in the Saudi Arabian hyperuricemia patients and their serum uric acid levels.

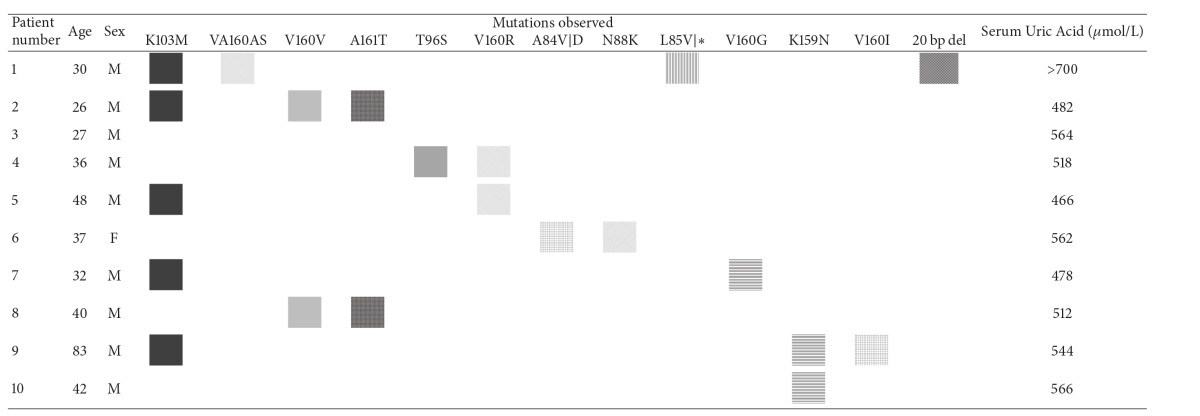

M: male; F: female.

**Table 2:** Amino acid location, substitutions, and predictions associated with HPRT gene in Saudi Arabian hyperuricemia patients.

Mutation	Location	Amino acid	Type	cDNA position	Sift prediction			Polyphen prediction			SNPnexus (Genetic association database)		
					Score	Prediction	Confidence	Score	Prediction	Phenotype	Association	GAD Id	Prevalence in patients
384A>T	Exon 3	Lys103Met	Nonsynonymous	475	0	damaging	high	0.997	damaging	Lesch-Nyhan syndrome	Y	126393	1, 2, 5, 7 & 9
614delTCG	Exon 6	ValAla160AlaSer	Peptide shift	646	N	N	N	N	N	Lesch-Nyhan syndrome	Y	126393	1
615C>G	Exon 6	Val160Val	Synonymous	647	N	N	N	N	N	Lesch-Nyhan syndrome	Y	126393	2, 8
616G>A	Exon 6	Ala161Thr	Nonsynonymous	648	0.08	tolerated	high	0.928	damaging	Lesch-Nyhan syndrome	Y	126393	2, 8
362A>T	Exon 3	Thr96Ser	Nonsynonymous	453	0.92	tolerated	high	0	tolerated	Lesch-Nyhan syndrome	Y	126393	4
613delG	Exon 6	Val160Arg	Frameshift: stop-gain	645	N	N	N	N	N	Lesch-Nyhan syndrome	Y	126393	4, 5
327insT	Exon 3	Ala84Val | Asp	Nonsynonymous|frameshift: stop-gain	418	0.34	tolerated	high	0	tolerated	Lesch-Nyhan syndrome	Y	126393	6
340T>A	Exon 3	Asn88Lys	Nonsynonymous	431	0.15	tolerated	high	0.015	tolerated	Lesch-Nyhan syndrome	Y	126393	6
329insG	Exon 3	Leu85Val | ∗	Nonsynonymous|frameshift: stop-gain	420	0.01	damaging	high	0.505	damaging	Lesch-Nyhan syndrome	Y	126393	1
614T>G	Exon 6	Val160Gly	Nonsynonymous	638	0	damaging	high	0.994	damaging	Lesch-Nyhan syndrome	Y	126393	7
612G/T	Exon 6	Lys159Asn	Nonsynonymous	644	0.3	tolerated	high	0.184	tolerated	Lesch-Nyhan syndrome	Y	126393	9, 10
613G>A	Exon 6	Val160Ile	Nonsynonymous	645	0.11	tolerated	high	0.107	tolerated	Lesch-Nyhan syndrome	Y	126393	9
20 bp deletion	Exons 5 and 6	—	Deletion	—	—	—	—	—	—	—	—	—	1

**Table 3:** Predicting the effect of the two mutations (K103M and V160G) on HPRT protein function using SNAP and PoPMuSiC.

Mutations	SNAP			PoPMuSiC	
nsSNP	Prediction	Reliability index	Expected accuracy	ΔΔ*G* (kcal/mol)	Prediction
K103M	Nonneutral	2	70%	−0.04	Stabilizing
V160G	Nonneutral	1	63%	3.23	Destabilizing

## References

[1] Roubenoff R (1990). Gout and hyperuricemia. *Rheumatic Disease Clinics of North America*.

[2] Tikly M, Bellingan A, Lincoln D, Russell A (1998). Risk factors for gout: a hospital-based study in urban Black South Africans. *Revue du Rhumatisme*.

[3] McGill NW (2000). Gout and other crystal-associated arthropathies. *Bailliere's Best Practice and Research in Clinical Rheumatology*.

[4] Emmerson B (1998). Hyperlipidaemia in hyperuricaemia and gout. *Annals of the Rheumatic Diseases*.

[5] Vuorinen-Markkola H, Yki-Järvinen H (1994). Hyperuricemia and insulin resistance. *Journal of Clinical Endocrinology and Metabolism*.

[6] Cordain L, Eaton SB, Sebastian A (2005). Origins and evolution of the Western diet: health implications for the 21st century. *American Journal of Clinical Nutrition*.

[7] Johnson RJ, Perez-Pozo SE, Sautin YY (2009). Hypothesis: could excessive fructose intake and uric acid cause type 2 diabetes?. *Endocrine Reviews*.

[8] Johnson RJ, Segal MS, Sautin Y (2007). Potential role of sugar (fructose) in the epidemic of hypertension, obesity and the metabolic syndrome, diabetes, kidney disease, and cardiovascular disease. *The American Journal of Clinical Nutrition*.

[9] Jinnah HA, de Gregorio L, Harris JC, Nyhan WL, O'Neill JP (2000). The spectrum of inherited mutations causing HPRT deficiency: 75 new cases and a review of 196 previously reported cases. *Mutation Research*.

[10] Edwards A, Voss H, Rice P (1990). Automated DNA sequencing of the human HPRT locus. *Genomics*.

[11] Kelley WN, Rosenbloom FM, Henderson JF, Seegmiller JE (1967). A specific enzyme defect in gout associated with overproduction of uric acid. *Proceedings of the National Academy of Sciences of the United States of America*.

[12] Jinnah HA, De Gregorio L, Harris JC, Nyhan WL, O'Neill JP (2000). The spectrum of inherited mutations causing HPRT deficiency: 75 new cases and a review of 196 previously reported cases. *Mutation Research—Reviews in Mutation Research*.

[13] Jinnah HA, Harris JC, Nyhan WL, O'Neill JP (2004). The spectrum of mutations causing HPRT deficiency: an update. *Nucleosides, Nucleotides and Nucleic Acids*.

[14] Rajendran V, Purohit R, Sethumadhavan R (2012). In silico investigation of molecular mechanism of laminopathy caused by a point mutation (R482W) in lamin A/C protein. *Amino Acids*.

[15] Purohit R, Rajendran V, Sethumadhavan R (2011). Relationship between mutation of serine residue at 315th position in *M. tuberculosis* catalase-peroxidase enzyme and Isoniazid susceptibility: an in silico analysis. *Journal of Molecular Modeling*.

[16] Sambrook J, Fritsch EF, Maniatis T (1989). *Molecular Cloning: A Laboratory Manual*.

[17] Ng PC, Henikoff S (2006). Predicting the effects of amino acid substitutions on protein function. *Annual Review of Genomics and Human Genetics*.

[18] Becker KG, Barnes KC, Bright TJ, Wang SA (2004). The genetic association database. *Nature Genetics*.

[19] Conrad DF, Andrews TD, Carter NP, Hurles ME, Pritchard JK (2006). A high-resolution survey of deletion polymorphism in the human genome. *Nature Genetics*.

[20] Hinds DA, Kloek AP, Jen M, Chen X, Frazer KA (2006). Common deletions and SNPs are in linkage disequilibrium in the human genome. *Nature Genetics*.

[21] Iafrate AJ, Feuk L, Rivera MN (2004). Detection of large-scale variation in the human genome. *Nature Genetics*.

[22] Locke DP, Sharp AJ, McCarroll SA (2006). Linkage disequilibrium and heritability of copy-number polymorphisms within duplicated regions of the human genome. *American Journal of Human Genetics*.

[23] McCarroll SA, Hadnott TN, Perry GH (2006). Common deletion polymorphisms in the human genome. *Nature Genetics*.

[24] Redon R, Ishikawa S, Fitch KR (2006). Global variation in copy number in the human genome. *Nature*.

[25] Sebat J, Lakshmi B, Troge J (2004). Large-scale copy number polymorphism in the human genome. *Science*.

[26] Sharp AJ, Locke DP, McGrath SD (2005). Segmental duplications and copy-number variation in the human genome. *American Journal of Human Genetics*.

[27] Tuzun E, Sharp AJ, Bailey JA (2005). Fine-scale structural variation of the human genome. *Nature Genetics*.

[28] Sherry ST, Ward MH, Kholodov M (2001). dbSNP: the NCBI database of genetic variation. *Nucleic Acids Research*.

[29] Frazer KA, Ballinger DG, Cox DR (2007). A second generation human haplotype map of over 3.1 million SNPs. *Nature*.

[30] Roy A, Kucukural A, Zhang Y (2010). I-TASSER: a unified platform for automated protein structure and function prediction. *Nature protocols*.

[31] Bromberg Y, Yachdav G, Rost B (2008). SNAP predicts effect of mutations on protein function. *Bioinformatics*.

[32] Dehouck Y, Grosfils A, Folch B, Gilis D, Bogaerts P, Rooman M (2009). Fast and accurate predictions of protein stability changes upon mutations using statistical potentials and neural networks: PoPMuSiC-2.0. *Bioinformatics*.

[33] Brooks BR, Brooks CL, Mackerell AD (2009). CHARMM: the biomolecular simulation program. *Journal of Computational Chemistry*.

[34] Tomita-Mitchell A, Ling LL, Glover CL, Goodluck-Griffith J, Thilly WG (2003). The mutational spectrum of the HPRT gene from human T cells in vivo shares a significant concordant set of hot spots with MNNG-treated human cells. *Cancer Research*.

[35] Duan J, Nilsson L, Lambert B (2004). Structural and functional analysis of mutations at the human hypoxanthine phosphoribosyl transferase (HPRT1) locus. *Human Mutation*.

[36] Fu R, Ceballos-Picot I, Torres RJ (2014). Genotype-phenotype correlations in neurogenetics: Lesch-Nyhan disease as a model disorder. *Brain*.

[37] Burkhart-Schultz KJ, Thompson CL, Jones IM (1996). Spectrum of somatic mutation at the hypoxanthine phosphoribosyltransferase (hprt) gene of healthy people. *Carcinogenesis*.

[38] Cariello NF, Scott JK, Kat AG, Thilly WG, Keohavong P (1988). Resolution of a missense mutant in human genomic DNA by denaturing gradient gel electrophoresis and direct sequencing using in vitro DNA amplification: HPRT(Munich). *American Journal of Human Genetics*.

[39] Corrigan A, Arenas M, Escuredo E, Fairbanks L, Marinaki A (2011). HPRT deficiency: identification of twenty-four novel variants including an unusual deep intronic mutation. *Nucleosides, Nucleotides and Nucleic Acids*.

[40] Hackman P, Hou S, Nyberg F, Pershagen G, Lambert B (2000). Mutational spectra at the hypoxanthine-guanine phosphoribosyltransferase (HPRT) locus in T-lymphocytes of nonsmoking and smoking lung cancer patients. *Mutation Research—Genetic Toxicology and Environmental Mutagenesis*.

[41] Mizunuma M, Yamada Y, Yamada K (2004). Disruption of the hypoxanthine-guanine phosphoribosyl-transferase gene caused by a translocation in a patient with Lesch-Nyhan syndrome. *Nucleosides, Nucleotides and Nucleic Acids*.

[42] Nguyen KV, Nyhan WL (2013). Identification of novel mutations in the human HPRT gene. *Nucleosides, Nucleotides and Nucleic Acids*.

[43] Podlutsky A, Österholm A, Hou S, Hofmaier A, Lambert B (1998). Spectrum of point mutations in the coding region of the hypoxanthine-guanine phosphoribosyltransferase (hprt) gene in human T-lymphocytes in vivo. *Carcinogenesis*.

[44] Podlutsky A, Hou S, Nyberg F, Pershagen G, Lambert B (1999). Influence of smoking and donor age on the spectrum of in vivo mutation at the HPRT-locus in T lymphocytes of healthy adults. *Mutation Research—Fundamental and Molecular Mechanisms of Mutagenesis*.

[45] Yamada Y, Yamada K, Nomura N (2010). Molecular analysis of two enzyme genes, HPRT1 and PRPS1, causing X-linked inborn errors of purine metabolism. *Nucleosides, Nucleotides and Nucleic Acids*.

[46] Willers I, Bolz H, Wehnert M, Gal A (1999). Eighteen novel mutations in patients with Lesch-Nyhan syndrome or partial hypoxanthine phosphoribosyltransferase deficiency. *Journal of Inherited Metabolic Disease*.

[47] Gibbs RA, Nguyen P, Edwards A, Civitello AB, Caskey CT (1990). Multiplex DNA deletion detection and exon sequencing of the hypoxanthine phosphoribosyltransferase gene in Lesch-Nyhan families. *Genomics*.

[48] Davidson BL, Chin SJ, Wilson JM, Kelley WN, Palella TD (1988). Hypoxanthine-guanine phosphoribosyltransferase. Genetic evidence for identical mutations in two partially deficient subjects. *The Journal of Clinical Investigation*.

[49] Sege-Peterson K, Chambers J, Page T, Jones OW, Nyhan WL (1992). Characterization of mutations in phenotypic variants of hypoxanthine phosphoribosyltransferase deficiency. *Human Molecular Genetics*.

[50] Yamada Y, Nomura N, Yamada K, Wakamatsu N (2007). Molecular analysis of HPRT deficiencies: an update of the spectrum of Asian mutations with novel mutations. *Molecular Genetics and Metabolism*.

[51] Tarle SA, Davidson BL, Wu VC (1991). Determination of the mutations responsible for the lesch-nyhan syndrome in 17 subjects. *Genomics*.

[52] Gibbs RA, Nguyen PN, McBride LJ, Koepf SM, Caskey CT (1989). Identification of mutations leading to the Lesch-Nyhan syndrome by automated direct DNA sequencing of in vitro amplified cDNA. *Proceedings of the National Academy of Sciences of the United States of America*.

[53] Tvrdik T, Marcus S, Hou S (1998). Molecular characterization of two deletion events involving Alu-sequences, one novel base substitution and two tentative hotspot mutations in the hypoxanthine phosphoribosyltransferase (HPRT) gene in five patients with Lesch-Nyhan syndrome. *Human Genetics*.

[54] Yamada Y, Goto H, Suzumori K, Adachi R, Ogasawara N (1992). Molecular analysis of five independent Japanese mutant genes responsible for hypoxanthine guanine phosphoribosyltransferase (HPRT) deficiency. *Human Genetics*.

[55] Ogasawara N, Stout JT, Goto H, Sonta S, Matsumoto A, Caskey CT (1989). Molecular analysis of a female Lesch-Nyhan patient. *Journal of Clinical Investigation*.

[56] Ogasawara N, Yamada Y, Goto H (1991). HPRT gene mutations in a female Lesch-Nyhan patient. *Advances in Experimental Medicine and Biology*.

[57] Yamada Y, Goto H, Yukawa T, Akazawa H, Ogasawara N (1994). Molecular mechanisms of the second female Lesch-Nyhan patient. *Advances in Experimental Medicine and Biology*.

[58] Yamada Y, Suzumori K, Tanemura M, Goto H, Ogasawara N (1996). Molecular analysis of a Japanese family with Lesch-Nyhan syndrome: identification of mutation and prenatal diagnosis. *Clinical Genetics*.

[59] Kamaraj B, Purohit R (2013). *In silico* screening and molecular dynamics simulation of disease-associated nsSNP in TYRP1 gene and its structural consequences in OCA3. *BioMed Research International*.

[60] Kumar A, Purohit R (2014). Use of long term molecular dynamics simulation in predicting cancer associated SNPs. *PLoS Computational Biology*.

[61] Kumar A, Rajendran V, Sethumadhavan R, Purohit R (2013). Molecular dynamic simulation reveals dam aging impact of RAC1 F28L mutation in the switch I region. *PLoS ONE*.

[62] Kumar A, Purohit R (2012). Computational investigation of pathogenic nsSNPs in CEP63 protein. *Gene*.

