# Supplementary material for: Novel Hypoxanthine Guanine Phosphoribosyltransferase Gene Mutations in Saudi Arabian Hyperuricemia Patients
Source: Biomed Res Int. 2014 Jul 9;2014:290325. doi: 10.1155/2014/290325 (PMC4119946; doi:10.1155/2014/290325)
Supplement: Supplementary file 1 — The supplementary tables and figures show the PCR conditions and primers used to amplify the 9 exonic regions of the Saudi Arabian hyperuricemia patients (Table 1), with energy minimization and molecular dynamic simulations performed for the substituted amino acids Lys103Met and Val160Gly at various minimization cycles using conjugate gradient method (Tables 2–4, Supplementary Figures 1–3). [file 290325.f1.zip › source/Supplementary_Table_1_2738_913312.docx]

| Primers | Sequence | Melting Temperature | Annealing Temperature ºC | Fragment Size (bp) |
| --- | --- | --- | --- | --- |
| HPRT 1F | CCTCCTCCTGAGCAGTCAGC | 53.8 | 62 | 229 |
| HPRT 1R | CGTGACGTAAAGCCGAACC | 53.1 |  |  |
| HPRT 2F | CCGGCCTGTTGTTTTCTTAC | 51.5 | 61 | 445 |
| HPRT 2R | AAGGCCCTCCTCTTTTATTTTT | 51.8 |  |  |
| HPRT 3F | CCTTATGAAACATGAGGGCAAAGG | 57.8 | 61 | 646 |
| HPRT 3R | TCTCACTGTAACCAAGTGAAATGAA | 52.5 |  |  |
| HPRT 4F | TAGCTAGCTAACTTCTCAAATCTTCTAG | 50.8 | 64 | 334 |
| HPRT 4R | ATTAACCTAGACTGCTTCCAAGGG | 54.2 |  |  |
| HPRT 5F | GGGTTGTTATGATGTGATTTGA | 49.1 | 61 | 192 |
| HPRT 5R | GAGGAATTTCTCTCCCTGGC | 51.9 |  |  |
| HPRT 6F | GACAGTATTGCAGTTATACATGGGG | 53.7 | 61 | 441 |
| HPRT 6R | CCAAAATCCTCTGCCATGCTATTC | 57.8 |  |  |
| HPRT 7-8F | CCCTGTAGTCTCTCTGTATG | 40.6 | 57 | 433 |
| HPRT 7-8R | TTATGAGGTGCTGGAAGGAG | 49.3 |  |  |
| HPRT 9F | TTCAAAAGATACACTCCCCAAAA | 52.4 | 57 | 383 |
| HPRT 9R | TTAGGAATGCAGCAACTGACA | 51.0 |  |  |

TABLE 1: Primers and PCR conditions used to amplify HPRT gene in Saudi Arabian Hyperuricemia patients.

Abbreviations: F, forward; PCR, polymerase chain reaction; R, reverse.
