# Supplementary figures and images for: Novel Hypoxanthine Guanine Phosphoribosyltransferase Gene Mutations in Saudi Arabian Hyperuricemia Patients
Source: Biomed Res Int. 2014 Jul 9;2014:290325. doi: 10.1155/2014/290325 (PMC4119946; doi:10.1155/2014/290325)

## Slide 1
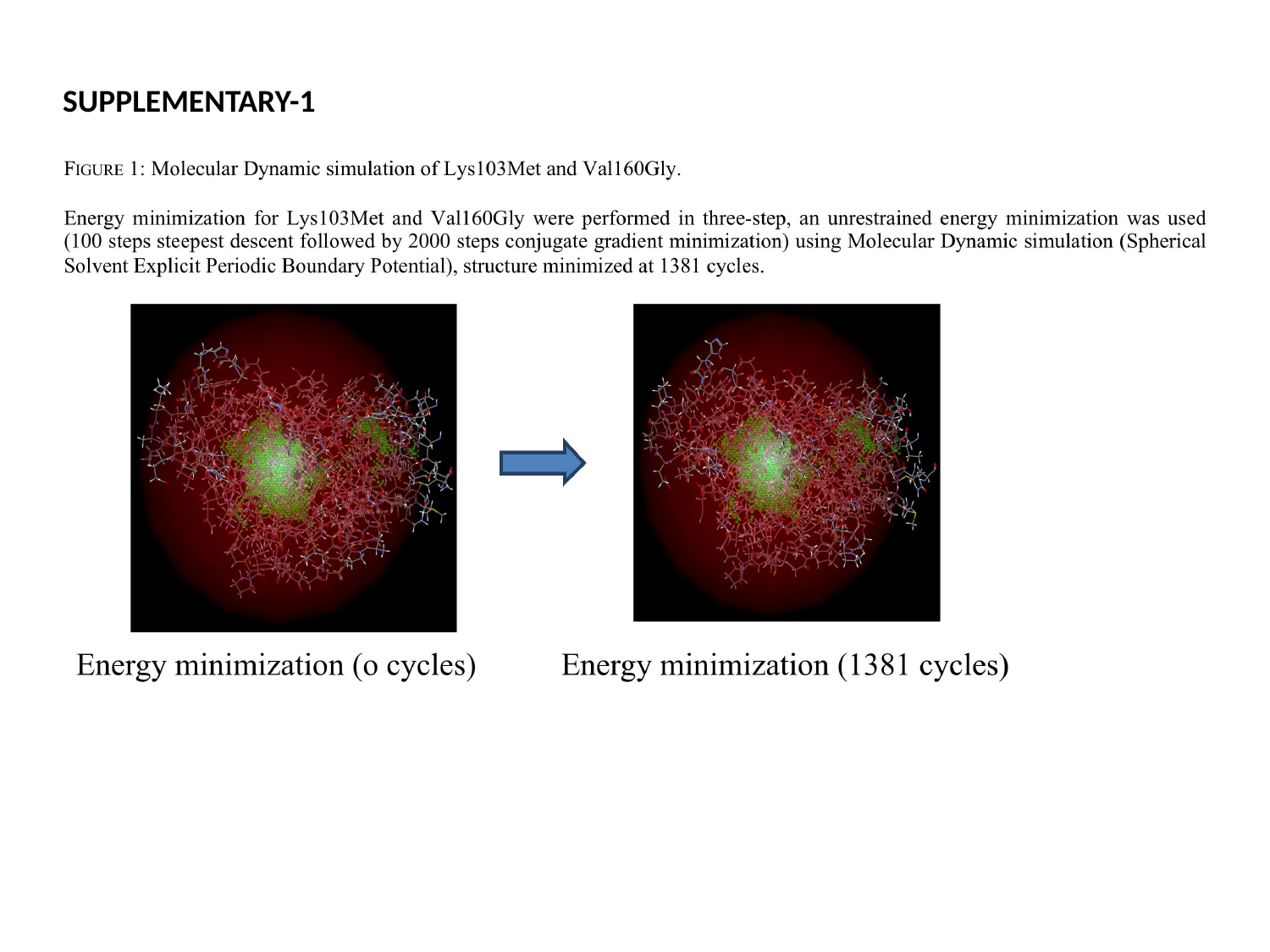

SUPPLEMENTARY-1

Supplement: Supplementary file 1 — The supplementary tables and figures show the PCR conditions and primers used to amplify the 9 exonic regions of the Saudi Arabian hyperuricemia patients (Table 1), with energy minimization and molecular dynamic simulations performed for the substituted amino acids Lys103Met and Val160Gly at various minimization cycles using conjugate gradient method (Tables 2–4, Supplementary Figures 1–3). [file 290325.f1.zip › source/Supplementary_Figure_1_2738_916433.pptx]

## Slide 1
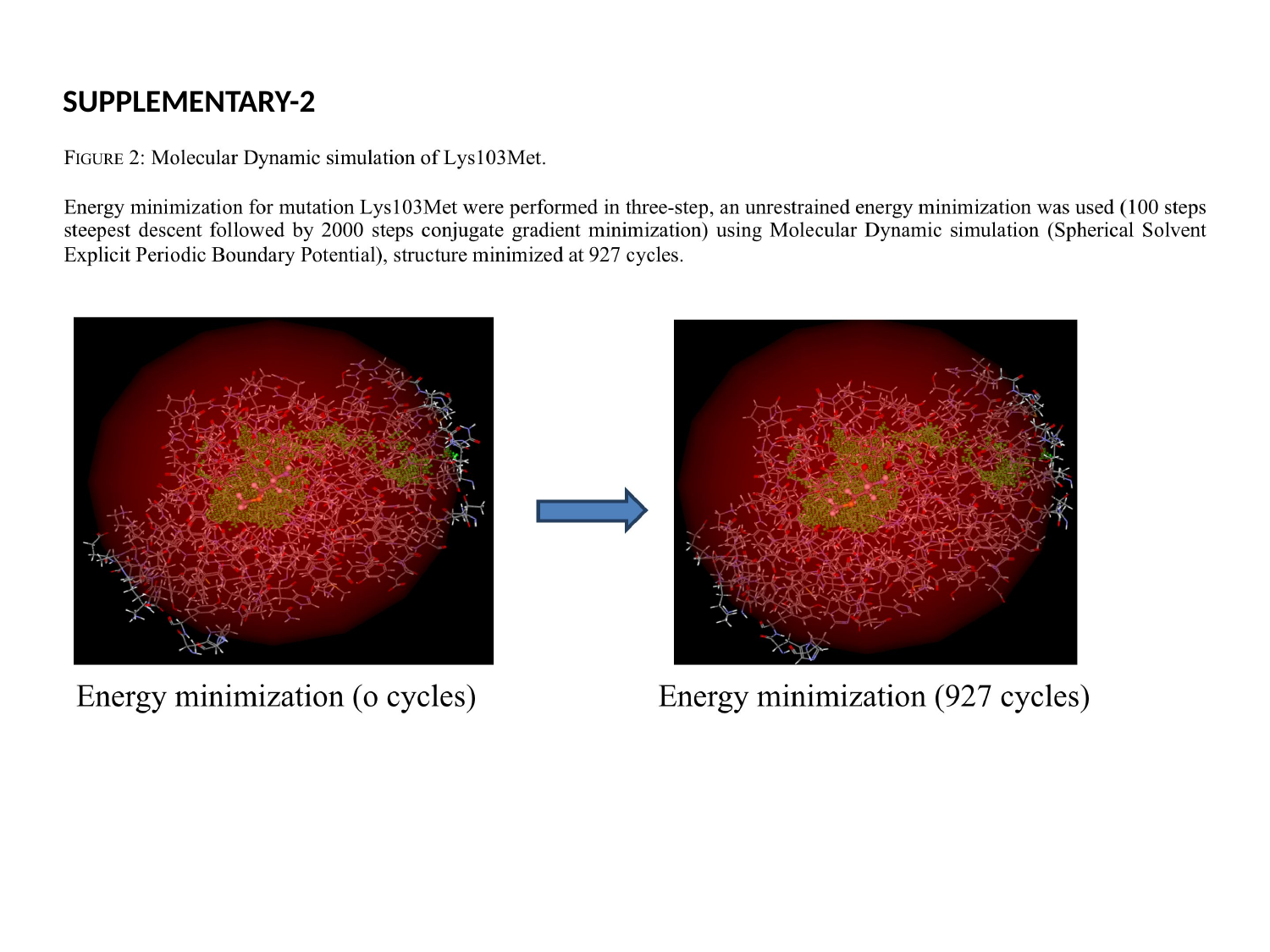

SUPPLEMENTARY-2

Supplement: Supplementary file 1 — The supplementary tables and figures show the PCR conditions and primers used to amplify the 9 exonic regions of the Saudi Arabian hyperuricemia patients (Table 1), with energy minimization and molecular dynamic simulations performed for the substituted amino acids Lys103Met and Val160Gly at various minimization cycles using conjugate gradient method (Tables 2–4, Supplementary Figures 1–3). [file 290325.f1.zip › source/Supplementary_Figure_2_2738_916434.pptx]

## Slide 1
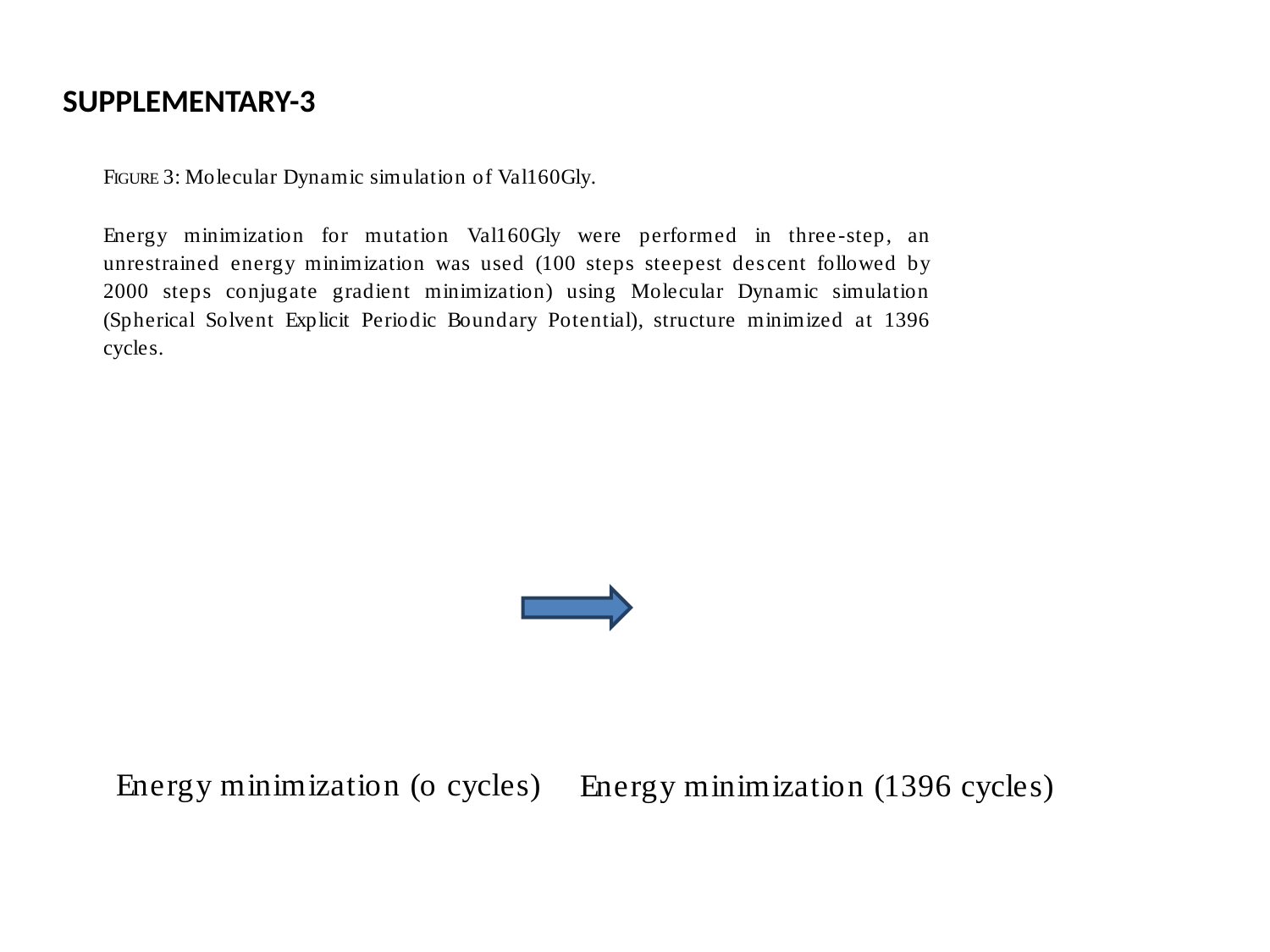

SUPPLEMENTARY-3

Supplement: Supplementary file 1 — The supplementary tables and figures show the PCR conditions and primers used to amplify the 9 exonic regions of the Saudi Arabian hyperuricemia patients (Table 1), with energy minimization and molecular dynamic simulations performed for the substituted amino acids Lys103Met and Val160Gly at various minimization cycles using conjugate gradient method (Tables 2–4, Supplementary Figures 1–3). [file 290325.f1.zip › source/Supplementary_Figure_3_2738_916435.pptx]
